# Mechanism of Hydrophobic Bile Acid-Induced Hepatocyte Injury and Drug Discovery

**DOI:** 10.3389/fphar.2020.01084

**Published:** 2020-07-16

**Authors:** Shizhang Wei, Xiao Ma, Yanling Zhao

**Affiliations:** ^1^ College of Pharmacy, Chengdu University of Traditional Chinese Medicine, Chengdu, China; ^2^ Department of Pharmacy, PLA General Hospital, Beijing, China

**Keywords:** cholestasis, bile acid, liver injury, mechanism, drug

## Abstract

Cholestatic liver disease is caused by the obstruction of bile synthesis, transport, and excretion in or outside the liver by a variety of reasons. Long-term persistent cholestasis in the liver can trigger inflammation, necrosis, or apoptosis of hepatocytes. Bile acid nuclear receptors have received the most attention for the treatment of cholestasis, while the drug development for bile acid nuclear receptors has made considerable progress. However, the targets regulated by bile acid receptor drugs are limited. Thus, as anticipated, intervention in the expression of bile acid nuclear receptors alone will not yield satisfactory clinical results. Therefore, this review comprehensively summarized the literature related to cholestasis, analyzed the molecular mechanism that bile acid damages cells, and status of drug development. It is hoped that this review will provide some reference for the research and development of drugs for cholestasis treatment in the future.

## Introduction 

Bile acid is an important component of bile, accounting for about 85% of the solid composition of bile, is the main metabolite of cholesterol metabolism, which can participate in the regulation of physiological function, such as cholesterol, sugar, and lipid metabolism (Song et al., 2015). After being synthesized in the liver, bile acid is secreted into the gallbladder through the bile duct, then enters the small intestine to participate in food digestion, and finally re-absorbs into the liver again. Another 5% of bile acids are excreted through feces (Martinot et al., 2017). Bile acids are mainly conjugated bile acids in the hepato-intestinal circulation and cannot pass through the cell membrane, so the metabolic process of bile acids requires the participation of a variety of metabolic enzymes, nuclear receptors and transporters, as well as the bile acids transport membrane system in the hepato-intestinal circulation (Trauner et al., 2017).

Cholestasis is an obstacle in the secretion and excretion of bile acid, which results in bile acid not flowing into the small intestine but flowing into the blood reversely. The toxic bile acid accumulated in the liver and systemic circulation for a long time can cause damage to the bile duct and liver cells, and severe cases can cause liver fibrosis and cirrhosis (Yan et al., 2017). Clinically, it is common in acute and chronic liver diseases such as primary sclerosing cholangitis (PSC), primary biliary cirrhosis (PBC), viral hepatitis, and drug-induced liver injury (de Vries and Beuers, 2017), and the main clinical manifestations are jaundice, pruritus, liver dysfunction, etc. (Wagner and Trauner, 2016).

Currently, numerous studies have investigated the pathogenesis of cholestasis. The mechanism of bile acid homeostasis is currently the hottest research hotspot. The key signaling pathways that regulate bile acid metabolism, such as bile acid synthesis-related targets farnesoid X receptor (FXR) (Farr et al., 2020), cholesterol 7-alpha hydroxy-lase (CYP7A1) (Chambers et al., 2019), short heterodimer partner (SHP) (Keitel et al., 2019), and oxysterol 12α-hydroxylase (CYP8B1) (Zhong et al., 2018), bile acid absorption-related targets Na+/taurocholate cotransporter (NTCP) (Li et al., 2019), bile acid transport targets bile salt export pump (BSEP), multidrug resistance-associated protein 2 (MRP2) (Wang et al., 2019), multidrug resistance-associated protein 3 (MRP3) (Song et al., 2019), and organic anion transporter family member 3A1 (OATP3A1) (Pan et al., 2018), and the bile acid detoxification-related targets pregnane X receptor (PXR) (Fan et al., 2019) and constitutive androstane receptor (CAR) (Shin and Wang, 2019), are the most concerned pathogenesis of cholestasis.

The discovery of the pathogenesis of cholestasis has pushed the research of cholestatic liver disease. Some small-molecule compound ligands are also found for the key targets regulating bile acid metabolism, which provides a clear direction for the discovery of cholestasis drugs and also provide better options for the treatment of patients with cholestasis. FXR agonist obeticholic acid (OCA) can significantly improve the symptoms and liver function of patients with cholestasis who do not respond to ursodeoxycholic acid (UDCA) (Bowlus et al., 2019). This gives us new hope for cholestatic liver diseases. However, some evidence has also found that OCA can also aggravate the symptoms of itching (Mayer et al., 2020). This leads us to rethink whether bile acid receptor drugs are the most promising treatments for patients with cholestatic liver disease. Besides, we also found that several studies have found that some non-bile acid receptor signaling pathways also play an important role in bile acid synthesis. However, the current research on non-nuclear receptor-targeted drugs is rare. So whether non-bile acid receptor ligands can be a new choice of drugs for cholestatic liver disease is also a question worthy of consideration. Therefore, this review focuses on the latest research progress about non-bile acid nuclear receptor mechanisms that regulate bile acid synthesis, hydrophobic bile acid toxicity mechanism, and current main drugs targeting bile acid receptor, hoping to provide some reference for the pathogenesis research and drug discovery research of cholestatic liver disease.

## Signals of Hydrophobic Bile Acid-Induced Liver Cell Death

Bile acids are composed of two types of bile acids: hydrophilic bile acids and hydrophobic bile acids. Hydrophobic bile acids, including glycocholic acid (GCA), cholic acid (CA), lithocholic acid (LCA), chenodeoxycholic acid (CDCA), and deoxycholic acid (DCA) are a major factor in inducing liver cell death (Thomas et al., 2008).

### “Hydrophobic Bile Acid-Death Ligand” Signals

**Figure 1:**
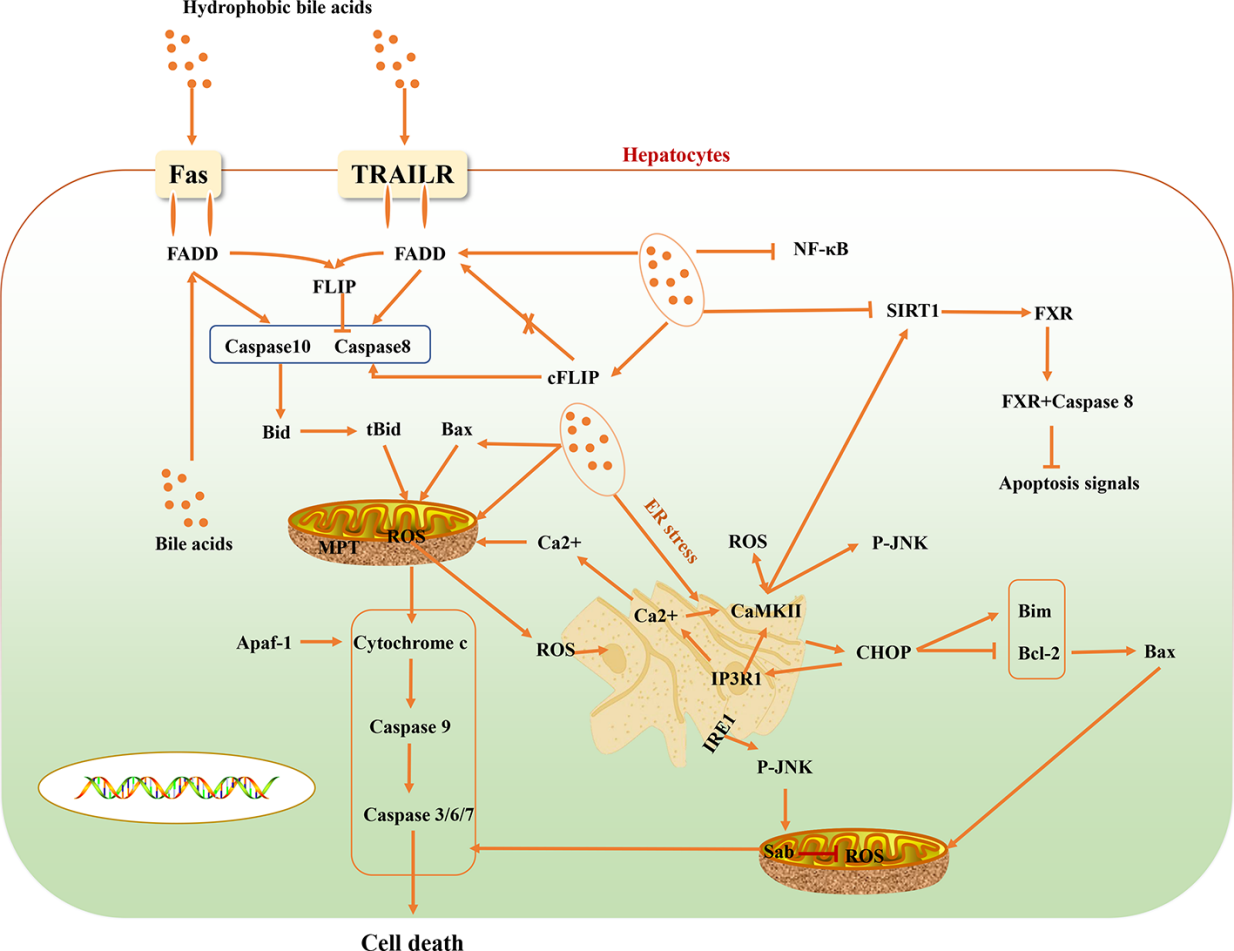
Multiple signaling pathways of hepatocyte death induced by hydrophobic bile acids.

Some reported evidence suggest that cholestasis-related hepatocyte apoptosis is related to death receptors (Sodeman et al., 2000). Activation of the TNF-related apoptosis-inducing ligand receptor (TRAILR) and Fas death receptor signaling pathway is an important pathway for hepatocyte apoptosis induced by bile acid. Bile acids activate Fas-related death signals in a ligand-dependent and -dependent hepatocyte apoptosis manner. Bile acid stimulates intracellular vesicles associated with the Golgi complex and the trans-Golgi network, and transfers Fas-containing vesicles to hepatocyte membranes, initiating a ligand-dependent death signaling pathway, while increasing Fas density on the surface of hepatocytes to making it more sensitive to Fas agonists. Bile acid-mediated apoptosis of hepatocytes not only activates ligand-independent death receptor oligomerization, but also regulates the sensitivity of death receptor-related signaling pathways. Death receptor-mediated apoptosis of hepatocytes is regulated by different apoptotic signals. On the death-inducing signaling complex (DISC), bile acid stimulates the phosphorylation of cFLIP to reduce the binding of two different isoforms of cFLIP long (cFLIP-L) and cFLIP short (cFLIP-S) to Fas-associated death domain (FADD) in DISC, and then reduce the recruitment of cFLIP to DISC, promoting the activation of caspases 8 and 10 (Higuchi et al., 2003; Higuchi and Gores, 2003). Activated caspases 8 and 10 cleave bid into tBid and enter it mitochondria with Bax to induce mitochondrial dysfunction and promote the release of cytochrome c. The released cytochrome c binds to apoptosis-activating factor-1 (Apaf-1) to promote the activation of Caspase9. And caspase9 further activates Caspase3/6/7, which eventually leads to liver cell death. Besides, bile acids can also directly cause Bax translocation into mitochondria, which can also lead to the release of cytochrome c and the downstream effectors of caspases signaling pathway. Bile acids can also stimulate mitochondrial respiratory chain to stimulate the production of reactive oxygen species (ROS) and cause mitochondrial membrane permeability transition (MPT), and release cytochrome c (Rodrigues et al., 1998; Higuchi et al., 2002) (**Figure 1**).

### “Intestinal Flora-Hydrophobic Bile Acid-Inflammation” Signals

Bile acids are mainly secreted by the liver, and 95% of them are reabsorbed into the ileum (Chavez-Talavera et al., 2019). Intestinal flora can regulate the body’s metabolism (Feng et al., 2019) and produce a large number of metabolites *in vivo*, which are important signal regulators and energy substrates (Swann et al., 2011). Intestinal flora modify bile acid molecules by debinding water, epimerization, and dehydroxylation. Bile salt hydrolase (BSH) in bacteria is the key enzyme for bile acid degradation in the intestinal tract (Joyce et al., 2014). It was found that Bacteroides, Lactobacillus, and Bifidobacterium can release primary bile acid by BSH (Swann et al., 2011). The primary bile acid is broken down into secondary bile acid (Sun et al., 2019), and esterified to make them more hydrophobic (Ridlon et al., 2016) by some other intestinal flora. Hydrophobic bile acid (such as DCA) in the secondary bile acid is cytotoxic and can cause liver cell damage (Xie et al., 2016) after reabsorption by liver. Moreover, intestinal flora can also modulate the synthesis of bile acids in an FXR dependent manner (Ridlon et al., 2013) and by influencing CYP7A1, Cyp8b1, CYP27A1 (Sayin et al., 2013).

Primary bile acids (DCA and CDCA) has been demonstrated to increase intestinal permeability (Raimondi et al., 2008). The intestinal LPS enters the blood circulation through the intestinal barrier and combines with the Toll-like receptor 4 (TLR4) receptor to further participate in the oxidative stress response and increase the overproduction of intracellular ROS in liver, reducing the activity of various antioxidant enzymes (Singh and Li, 2012; Chassaing et al., 2014). LPS can also induce macrophages to secrete IL-1, TNF-α to promote the release of adhesion molecules, stimulate the inflammatory response, and cause neutrophils to produce excess RO (Weber-Mzell et al., 2006). Hydrophobic bile acid is also a factor that stimulates inflammation, which can activate the production of inflammatory mediators (Allen et al., 2010). For example, CDCA and DCA up-regulate the expression of early growth response gene-1 (Egr-1) by activating the epithelial growth factor receptor (EGFR) to cause the production of vascular endothelial cell adhesion molecule-1 (VCAM-1), IL-1β, and IL-10 in liver cells (Yan et al., 2000). The pro-inflammatory factors produced by hepatocytes further stimulate and activate a variety of inflammatory cells, such as macrophages and neutrophils, to increase the degree of the inflammatory response in liver (Wintermeyer et al., 2009).

### “Nuclear Receptor-Apoptotic Protein” Signals

FXR can also regulate the synthesis of bile acids in the process of cholestasis through negative feedback regulation (Sinal et al., 2000). Unlike cFLIP, FXR in the cytoplasm does not directly inhibit the activation of caspase 8, but binds to caspase 8 to prevent the activation and conduction of apoptotic signals in a ligand-independent pathway. This is the fact that FXR agonists do not effectively improve the death receptor-induced hepatocyte death. Therefore, FXR in the liver cytoplasm can inhibit the over activation of caspase 8 by cooperating with cFLIP (Mattisson et al., 2017). However, in the process of liver injury, high levels of TRAIL, TNFα, and FasL in the blood circulation rapidly reduced the expression level of FXR before the activation of apoptosis signal, indicating that the decrease of FXR in the hepatocyte is the primary condition for the activation of apoptosis signal. NAD-dependent protein deacetylase sirtuin-1 (SIRT1) is a member of the silent information regulatory protein family and NAD-dependent deacetylase, which can affect many biological processes, including inflammation, glycolipid metabolism, and so on (Kulkarni et al., 2016). In the pathological state of cholestasis, toxic bile acids such as taurodeoxycholic acid (TDCA), taurocholic acid (TCA), and DCA can reduce the expression level of SIRT1 in hepatocytes (Zhao et al., 2019). SIRT is also the transcription regulator of FXR, and the FXR Lys217 is the main deacetylation binding site of FXR regulated by SIRT1 (Kemper et al., 2009). It can regulate the activity of FXR through deacetylation protein and histone (Purushotham et al., 2012). In addition, in mammals, peroxisome proliferator-activated receptor-γ coactivator 1α (PGC-1α) can be activated by SIRT1 through deacetylation and regulate the activity of FXR (Purushotham et al., 2012) (**Figure 1**).

### “Hydrophobic Bile Acid-Mitochondrial” Signals

Hepatocytes are rich in mitochondria. While producing adenosine triphosphate (ATP), mitochondria are also the main source of ROS, so hepatocytes are also the main target of ROS attack (Czaja, 2007). Under pathological conditions, excessive bile acids interfere with the mitochondrial respiratory complex and the electronic chain transmission process to decouple the oxidative respiratory chain, resulting in the generation of a large number of ROS. The ROS further stimulated mitochondrial permeability transition pore (MPTP) to leads to an irreversible open state, which in turn causes a large amount of high-molecular substances in the cytoplasm to enter the mitochondria, triggers mitochondrial hypertonicity and swells, and causes the mitochondrial membrane damage, ATP hydrolysis, and the release of Smac/DIABLO, cytochrome C, apoptosis-inducing factor (AIF) is released, eventually leading to hepatocyte apoptosis (Yerushalmi et al., 2001). Excess ROS can also increase the synthesis of active oxygen clusters in mitochondria by oxidation of antioxidants in mitochondria, further amplifying oxidative stress response. Simultaneously, excessive ROS can damage the dynamic balance of mitochondrial fusion and division related proteins and induce apoptosis (Wasiak et al., 2007).

On the other hand, excessive ROS can cause mitochondrial membrane depolarization, release cytochrome c to cytoplasm, activate caspase cascade, and induce apoptosis through caspase-3 related signaling pathway (Paradies et al., 2009). After a mitochondrial injury, glutamic oxalacetic transaminase (AST) in mitochondria will be released to the cytoplasm, and into the blood circulation (Perez et al., 2006).

### “Hydrophobic Bile Acid-Endoplasmic Reticulum” Signals

Hydrophobic bile acids can release calcium ions into the cytoplasm by inducing endoplasmic reticulum stress (ERS). The increased concentration of Ca^2+^ causes mitochondria to generate and release a large number of ROS, while the high level of ROS in the cytoplasm causes the increase of Ca^2+^ concentration. Moreover, hydrophobic bile acids directly stimulate mitochondria to release ROS and excessive ROS in the cytoplasm induces Ca^2+^ in the endoplasmic reticulum to enter the cytoplasm, and further stimulates mitochondria to produce excess ROS, causing a vicious cycle of oxidative stress in liver cells. All ERS sensors, including IRE1, are activated when there is an imbalance between the ER unfolded protein and chaperone protein (Tabas and Ron, 2011). ERS can activate JNK through IRE1 (Urano et al., 2000). JNK combines with Sab on mitochondria to inhibit mitochondrial respiration and ROS production (Win et al., 2014). C/EBP homologous protein (CHOP) is rarely expressed in the physiological state, but is expressed in large amounts when ERS occurs. ER oxidase 1α (ERO1α) is the direct target of ER-dependent oxidative stress induced by CHOP (Marciniak et al., 2004). ERO1α increases the level of Ca^2+^ in the cytoplasm by activating the ER calcium release channel IP3R1 (Li et al., 2009).

The role of CaMKII in ERS-induced apoptosis may be a part of the positive feedback amplification loop (Li et al., 2009). ROS in the cytoplasm can lead to the activation of Ca^2+^-dependent CaMKII (Biswas et al., 1999; Pizzo and Pozzan, 2007; Erickson et al., 2008). More importantly, another important downstream signal of chop CaMKII signaling pathway is the STAT1 signaling pathway (Li et al., 2010). When ERS occurs, another important mechanism of apoptosis induced by CHOP is the inhibition of the survival-promoting protein Bcl-2 (McCullough et al., 2001; Fu et al., 2010). Bcl-2 inhibits mitochondrial permeabilization and apoptosis through BH3 only proteins (including Bax, Bad, and Bim). Some studies have proved that Bim plays an important role in the apoptosis induced by ERS mediated by CHOP, and found that Bim knockout mice have a protective effect on apoptosis, and ERS increased the expression level of Bim (Puthalakath et al., 2007). Another study also found that there was an increase in the expression of Bax, which was dependent on CHOP, in ERS (Santos et al., 2009) (**Figure 1**).

## Main Bile Acid Receptor Drugs

### FXR Agonists

Farnesoid X receptor (FXR) is a bile acid nuclear receptor, which is highly expressed in liver and intestinal tissues, and plays an important role in the synthesis, absorption, metabolism, transport, and excretion of bile acids. FXR target genes, including SHP, BSEP, I-BABP, CYP3A4, SULT2A1, UGT2B4, etc. negatively regulate the uptake and synthesis of bile acids, and positively regulate genes responsible for bile acid excretion and detoxification (Fiorucci and Baldelli, 2009). The unconjugated bile acids produced in hepatocytes are mainly detoxified by the liver detoxification enzyme, such as CYP3A4, UGT1A1, and SULT2A1, to form bile acids with high water solubility, which are eliminated by the kidneys (Guengerich, 2001; Fujiwara et al., 2012). The main nuclear receptor of CYP3A4 is FXR. Therefore, FXR can not only regulate the synthesis of bile acids by feedback, but also accelerate the excretion of bile acids by increasing the hydrophilicity of hydrophobic bile acids. The FXR agonist OCA has been used clinically for the treatment of PBC. Studies have shown that it significantly improves the levels of serum ALP, ALT, AST of PBC patients. And, the long-term effect is good, but there are certain side effects (Kowdley et al., 2018). GW4064 is a non-steroidal FXR agonist. Animal experiments show that it can induce SHP in a FXR-selectively dependent manner, thereby reducing the expression of CYP8B1 and CYP7A1, and up-regulating the expression of NTCP (Moscovitz et al., 2016). WAY-362450 is also a highly selective FXR agonist, which can increase the expression of FXR, reduce the expression of CYP8B1 and CYP7A1 proteins, and reduce liver damage (Wu et al., 2014). In an ethanol-induced cholestatic liver injury study, it was found that intestinal FXR agonist Fexaramine can activate FXR, up-regulate the expression of SHP protein, reduce the expression of CYP7A1, and then reduce serum ALT levels in mice (Hartmann et al., 2018). As an FXR agonist, LJN 452 is more selective than GW4060, has better safety and tolerability in healthy volunteers, and is already undergoing phase II clinical trials in PBC patients (Tully et al., 2017). Ly2562175 is also a highly selective FXR agonist. Experiments have found that it has a significant effect on regulating blood lipids. It can reduce TG and LDL levels, and increase HDL levels. However, the indicators for regulating bile acid have not been evaluated (Genin et al., 2015). As a synthetic FXR agonist, GS-9674 mainly activates the expression of FGF19 by activating FXR on intestinal epithelial cells. FGF19 enters the liver to exert an inhibitory effect on bile acid synthesis. GS-9674 is currently in a phase II clinical trial for the treatment of PSC (Khanna and Jones, 2017).

**Figure 2:**
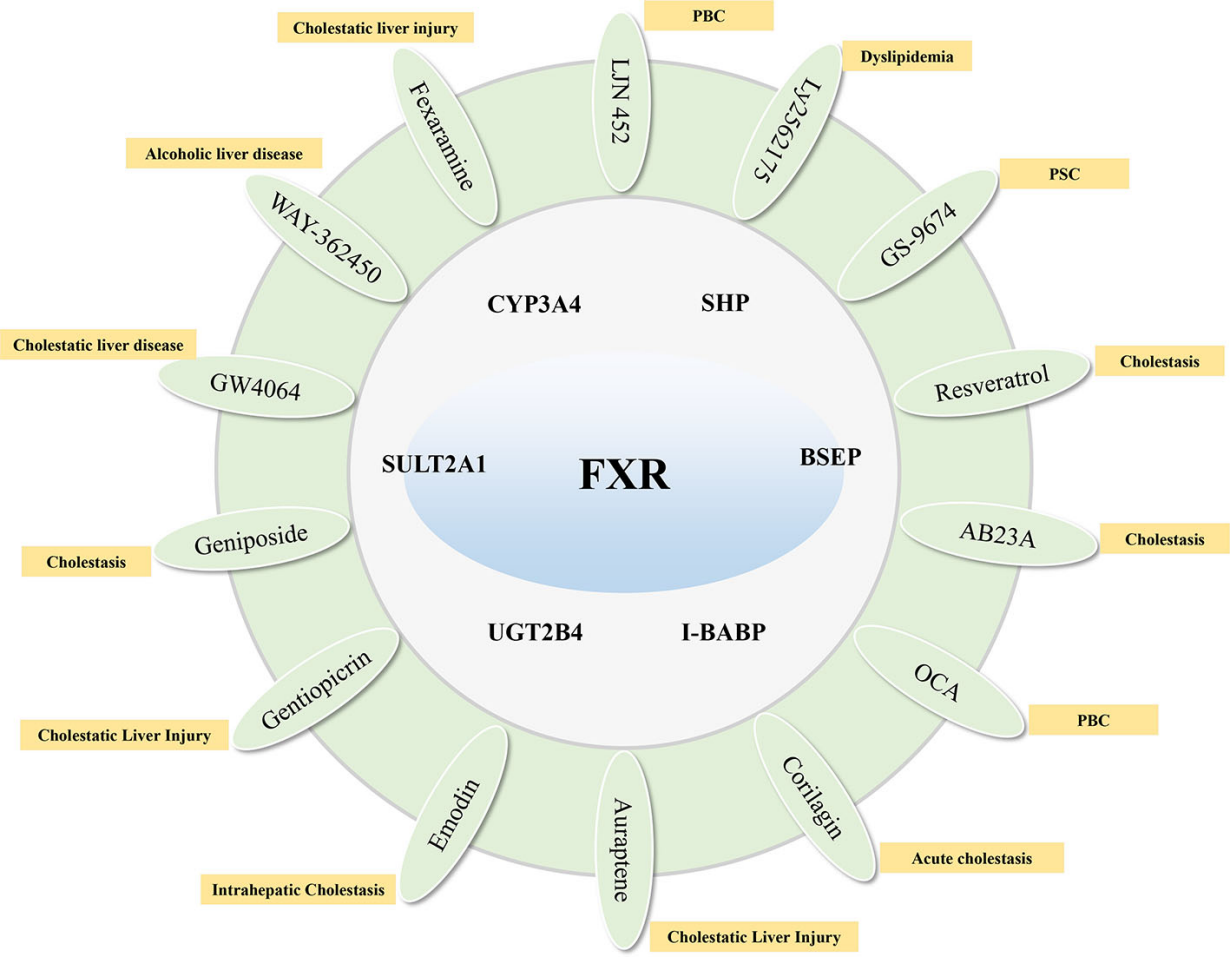
Compounds that target FXR to activate their target genes and related cholestatic liver diseases.

Traditional Chinese medicine (TCM) and its active ingredients also have significant advantages in the treatment of cholestasis. It has been proved that geniposide, an effective component of *Gardenia jasminoides*, can reduce the synthesis of bile acid by activating FXR, SHP, and OST β and reducing the expression of CYP7A1, Cyp8b1, and CYP27A1 (Wang et al., 2017). Resveratrol, one of the effective components of *Polygonum cuspidatum*, can regulate the bile acid homeostasis by inducing the expression of FXR and up-regulating the expression of BSEP, NTCP, and MRP2, thus reducing cholestasis (Ding et al., 2018). Applying a mouse model of cholestasis, it was found that the effective ingredient of *Alisma orientale* B23-acetate (AB23A) can activate bile acid synthesis negative feedback FXR signal, and then promote bile acid efflux and regulate bile acid metabolism (Meng et al., 2015). Corilagin, as one of the active ingredients of *Erodium stephaniahum*, can significantly improve serum liver function indexes of cholestasis rats, and regulate antioxidant and anti-inflammatory mechanisms (Jin et al., 2013). It can also activate FXR, SHP1, SHP2, UGT2B4, BSEP, MRP2, and SULT2A1 expression, down-regulate CYP7A1 and NTCP protein expression to improve cholestasis (Yang et al., 2018). Studies have shown that auraptene, the active ingredient in *Citrus reticulata*, reduces bile acid synthesis by activating the expression of FXR to reduce CYP7A1 and CYP8B1, and increases bile acid transporters (such as BSEP and MRP2) to increase bile acid transport (Gao et al., 2017). Emodin in *Rheum palmatum* can up-regulate the gene and protein expression of BSEP, FXR1, and FXR2 by activating the FXR/BSEP signaling pathway to reduce liver injury (Xiong et al., 2019). In addition, gentiopicrin, the main active ingredient in *Gentiana macrophylla*, can improve cholestatic liver injury by up-regulating the expression of FXR and MRP4 and reducing the expression of CYP7A1 (Han et al., 2018) (**Figure 2**).

### PXR Agonists

PXR is mainly responsible for regulating detoxification-related metabolic enzymes in the liver and small intestine, promoting the degradation of bile acids, and reducing the synthesis of bile acids (Kliewer and Willson, 2002; Jurica et al., 2016). The target genes for PXR include Cyp3a11 (Yamasaki et al., 2018), CYP7A1, CYP8B1, Cpy2b10, Oatp1a4, Oatp4 (Staudinger et al., 2001; Pavek, 2016), CYP3A4, MDR1 (Geick et al., 2001), MRP2 (Kast et al., 2002), MRP3 (Teng et al., 2003), SULT2A1 (Fang et al., 2007), UGT1A1/3/4 (Gardner-Stephen et al., 2004; van Dijk et al., 2015). In addition to inducing the bile acid efflux system, PXR can also activate the bile acid and bilirubin detoxification system (Jung et al., 2006; Fiorucci and Baldelli, 2009). Studies have shown that the activation of PXR can significantly reduce the bile acid synthesis by inhibiting the expression of CYP7A1, and the activation of PXR by FXR further enhances this effect (Staudinger et al., 2001).

Rifampin, an activator of PXR, promotes bilirubin excretion by inducing expression of SULT2A1, UGT1A1, and MRP2, and accelerates bile acid metabolism by increasing expression of CYP3A4 (Marschall et al., 2005; Li et al., 2013). Moreover, it can improve pruritus symptoms of patients with cholestasis (European Association for the Study of the 2009) and liver function biochemical indicators in PBC patients (Bachs et al., 1989). Rifampin is safe for up to 2 weeks (Khurana and Singh, 2006). However, atorvastatin as a PXR agonist cannot improve cholestasis in patients with PBC (Stojakovic et al., 2007).

**Figure 3:**
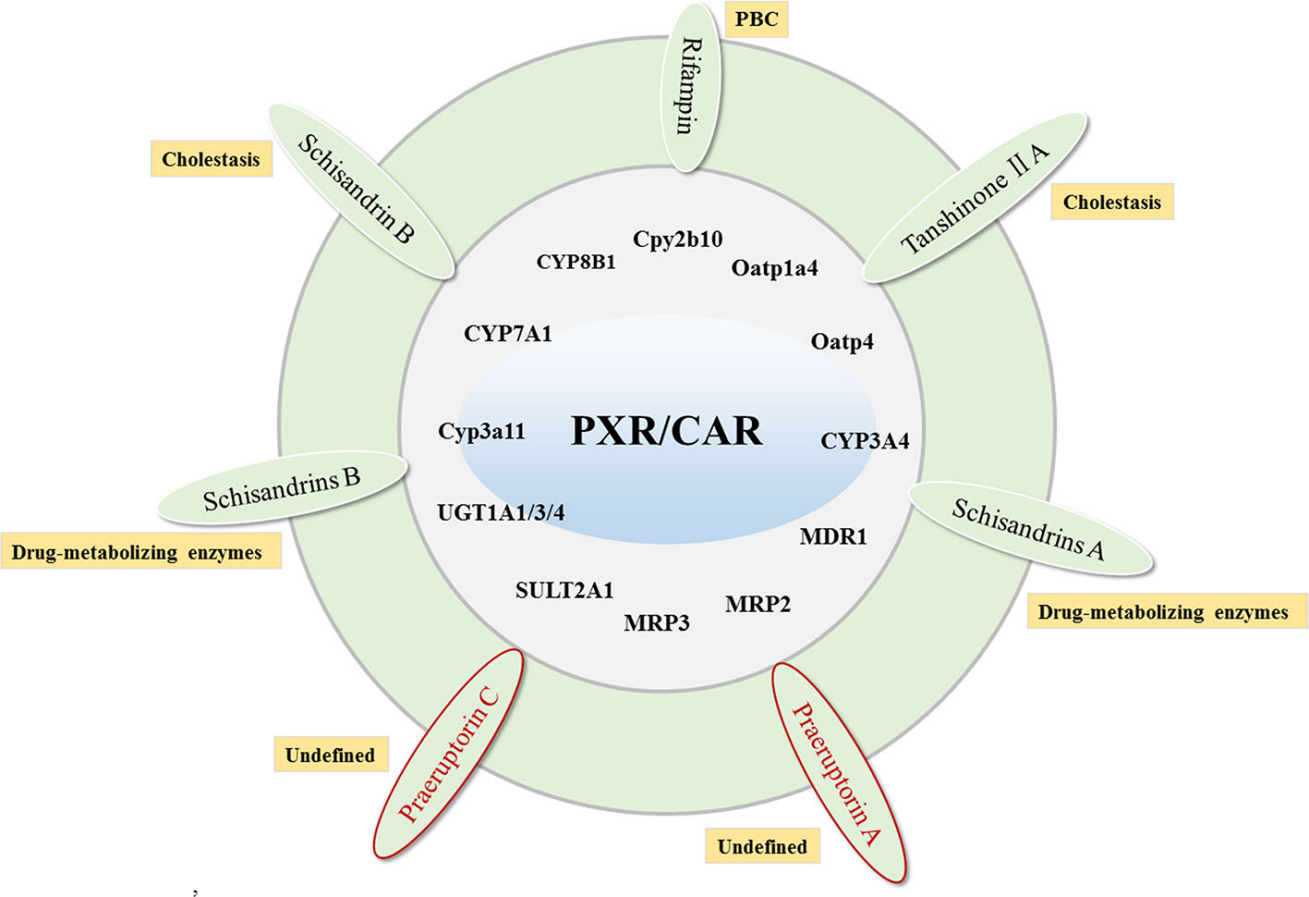
The compounds that target PXR/CAR to activate their target genes and related cholestatic liver diseases. Red labeled drugs are compounds that activate both PXR and CAR.

There are also some ingredients in herbs that can reduce cholestasis by activating the expression of PXR. Schisandrin B in *Schisandra chinensis* can reduce the bile acid levels in lithocholic acid-induced mice by activating PXR to induce the expression of Ugt1a1, Cyp3a11, and Oatp2 (Zeng et al., 2017). Tanshinone IIA is also a PXR agonist with significant liver protection. It is capable of preventing ANIT-induced cholestatic liver injury by improving the expression of CYP3A4, and simultaneously up-regulating the expression of Cyp3a11, Cyp3a13, and Mdr1 (Zhang X. et al., 2015). Schisandrins A, Schisandrins B, and Schisandrol B from *Schisandra chinensis* have also been reported to activate PXR expression in primary hepatocytes. Among them, Schisandrins A is also the main active ingredient in Wuzhi Tablets for the treatment of intrahepatic cholestasis (Mu et al., 2006; Zeng et al., 2016). Interestingly, due to the similarities in structural and functional between CAR and PXR, some active ingredients in herbs can activate both the targets simultaneously. Studies have reported that the praeruptorin A and C in *Peucedanum praeruptorum* can simultaneously activate the expression of PXR and CAR and the expression of CYP3A4 (Huang et al., 2013; Zhou et al., 2013) (**Figure 3**).

### CAR Agonists

CAR is one of the members of the orphan nuclear receptor superfamily. Its function and structure are very similar to PXR and are mainly expressed in liver and intestine. Studies have demonstrated that the activation of CAR can regulate UGT1A1, organic anion transporter SLC21A6, and MRP2 to accelerate the metabolism of bilirubin (Huang et al., 2003). More importantly, The CAR knockout mice cannot induce UGT1A1 expression, and also make mice more sensitive to toxic bile acids (Barbier et al., 2003). CAR shares common target genes with PXR and FXR, indicating that in addition to FXR agonists, CAR agonists may also be a potential treatment for cholestasis. CAR can activate CYP2B, CYP3A4, Sult2a1, UGT1A, MRP2 Mrp3, and MRP4 to induce bile acid excretion (Fiorucci and Baldelli, 2009) and resist hydrophobic bile acid-induced liver toxicity. At present, CAR agonist research is relatively rare. In addition to the praeruptorin A and C mentioned above, which can improve cholestasis by activating CAR, studies have found some CAR natural product agonists, such as berberine (Zhang et al., 2016), Arecoline (Ling et al., 2014), artemisinin (Burk et al., 2005), and 6,7-dimethylesculetin(Huang et al., 2004), etc. (**Figure 3**), but these CAR agonists have not been proven effective for cholestatic liver disease.

## FXR-PXR/CAR and FXR-Non-Bile Acid Nuclear Receptor Targets Coactivator

From the above, FXR, as a key target for regulating bile acids, has consistently been a promising target for the treatment of cholestatic liver disease. However, OCA targeting FXR has not shown satisfactory results in the clinical treatment of cholestatic liver disease. This leads us to think differently about drug development strategies that only intervention in bile acids and FXRs. It is well known that PXR and CAR are similar in structure and function, which can regulate the transcription and translation of bile acid metabolism enzymes and transporters related to bilirubin clearance, and play a detoxifying role in the liver (Kakizaki et al., 2011). Therefore, we speculate that drugs that can simultaneously regulate FXR and PXR/CAR may be more promising for the treatment of cholestatic liver disease. In addition, non-bile acid receptor also plays a key role in bile acid synthesis and bile acid-induced liver cell damage. Therefore, we also propose that drugs that can simultaneously regulate FXR and non-bile acid receptors are also important directions for drug development in the treatment of cholestatic liver disease. At present, some related compounds have been found. For example, Geniposide can simultaneously regulate the expression of FXR, PXR, NF-κB, Bax, and Bcl-2, and has a great effect on various liver diseases such as cholestasis and liver inflammation (Rong et al., 2017; Wang et al., 2017; Hu et al., 2018). Swertiamarin has been found to have a significant effect on improving cholestasis. It can simultaneously regulate FXR, PXR, bile acid transporters Mrp3, Mdr1, and Mrp4 and detoxification enzymes (Cyp3a, Ugt2b, Sult2a1, and Gsta1), increase the water solubility of hydrophobic bile acids, remove the combined bile acids (Feng et al., 2015; Zhang L. et al., 2015). Formononetin can improve cholestasis through Sirt1-FXR signal pathway and alleviate liver inflammation through JNK inflammatory signal pathway (Yang et al., 2019). In addition, formononetin can decrease acetaminophen induced hepatotoxicity by increasing Nrf2 activity (Jin et al., 2017). Resveratrol has a therapeutic effect on ANIT induced cholestasis by regulating the FXR pathway, and it also has a very good improvement on non-FXR target genes, such as liver inflammatory factors TNFα, IL-6, and IL-1β, as well as oxidation factor COX-2 (Ding et al., 2018). In addition, resveratrol can also regulate the PI3K-Akt signal pathway (Shu et al., 2020). It can be concluded that there are few researches on this kind of coactivators, and the only existing researches are still in the stage of laboratory research, which also puts forward a longer-term requirement for future drug development of cholestatic liver disease.

## Conclusions

The pathogenesis of cholestasis has been studied relatively clearly, which has provided strong support for the drug development of cholestatic liver disease. However, at present, very few drugs are used clinically for the treatment of cholestatic liver disease. After decades of effort, UDCA has been clinically preferred for the treatment of cholestatic liver disease (Wunsch et al., 2014; Ma et al., 2016), but some patients do not respond. With an in-depth understanding of the pathogenesis of cholestasis, nuclear receptors have been discovered to play a key role in bile acid metabolism. And bile acid receptor agonists are recognized as the most promising drugs for the treatment of cholestatic liver diseases, such as FXR agonist OCA, which can significantly improve patients who do not respond to UDCA. This gives us a temporary glimmer of hope. However, the side effects aggravating the pruritus symptoms in patients with PBC have led us to rethink the development of therapeutic drugs for cholestatic liver disease. It is well known that, while regulating the bile acid synthesis with FXR as the key target, PXR and CAR regulate the bile acid metabolism enzymes and transporters related to bilirubin clearance, and play a detoxifying role in the liver. In addition, non-bile acid receptor targets also play a key role in bile acid synthesis and bile acid-induced liver cell damage. Therefore, we speculate that drugs that can simultaneously regulate FXR and PXR/CAR or FXR and non-bile acid receptor targets may be more promising for the treatment of cholestatic liver disease. It is worth mentioning that due to the strong hepatotoxicity of hydrophobic bile acids, the research and development of drugs that directly target the activation of hepatocyte detoxification enzymes (CYP3A4, UGT1A1, and SULT2A1) to accelerate the metabolism of hydrophobic bile acids to hydrophilic bile acids is also a very promising strategy for the development of drugs for the treatment of cholestasis. However, there are few studies on this research strategy, and the existing researches are still in the laboratory research stage, which puts forward longer-term requirements for the future drug development of cholestatic liver disease.

## Author Contributions

YZ designed the research framework and direction. SW wrote the paper. XM helped to organize the literature or provide writing ideas.

## Funding

This work is financially supported by grants from the National Natural Science Foundation of China (81874365).

## Conflict of Interest

The authors declare that the research was conducted in the absence of any commercial or financial relationships that could be construed as a potential conflict of interest.
